# Burden of migraine in a Kuwaiti population: a door-to-door survey

**DOI:** 10.1186/s10194-017-0814-2

**Published:** 2017-10-13

**Authors:** Jasem Yousef Al-Hashel, Samar Farouk Ahmed, Raed Alroughani

**Affiliations:** 1 Neurology Department, Ibn Sina Hospital, P.O. Box 25427, 13115 Safat, Kuwait; 2 Faculty of Medicine, Kuwait University, P.O. Box 24923, 13110 Safat, Kuwait; 3 Neuropsychiatry department, Faculty of Medicine, Al-Minia University, P.O. Box 61519, Minia City, Minia 61111 Egypt; 4 Division of Neurology, Department of Medicine, Amiri Hospital, Sharq, Kuwait

**Keywords:** Epidemiology, Prevalence, Migraine, Burden, Kuwait

## Abstract

**Background:**

Migraine prevalence and disability imprints on Kuwaiti population are underreported. We aimed to measure the prevalence of migraine and to assess its burden in Kuwait.

**Methods:**

A cross-sectional community-based study was conducted which included biologically unrelated Kuwaiti adult population aged 18–65 years. They were randomly recruited from all six governments of Kuwait using stratified multistage cluster sampling. Trained interviewers visited the samples in door-to-door approach. The Headache-Attributed Restriction, Disability, and Social Handicap and Impaired Participation (HARDSHIP) questionnaire was used to collect the data. Demographic enquires were followed by diagnostic and disability questions.

**Results:**

A total of 15,523 subjects were identified; of whom 3588 (23%) were diagnosed as episodic migraine and 845 (5.4%) as chronic headache. Prevalence of episodic migraine was 31.71% in female versus 14.88% in males (*P* < 0.01) with a mean age of 34.56 ± 10.17 years. Most of migraine cohort (64.4%) sought medical advice with respect to their migraine headaches and the majority (62.4%) were seen by general practitioners (GPs) while 17.2% were assessed by neurologists and 3.7% was seen by other specialties. Tension type headache and sinus-related headaches were diagnosed in 8.9% and 2.1% of migraine subjects respectively. The majority (94.6%) of migraine subjects used symptomatic drugs for headache attacks, whereas 39.9% were taking preventive medication. In the preceding 3 months to the survey, subjects with episodic migraine had lost a mean of 1.97 days from their paid work or school attendance compared to 6.62 days in chronic headache sufferers (*P* < 0.001). Additionally, subjects with episodic migraine lost a mean of 1.40 days from household work compared to 5.35 days in subjects with chronic headache (*P* < 0.001). Participants with episodic migraine and chronic headache missed a mean of 2.81 and 3.85 days on social occasions, in the preceding 3 months (*P* < 0.001).

**Conclusions:**

Migraine in Kuwait is highly prevalent and it has a significant impact on activity of daily living, schooling/ employment and social occasions of patients. Accurate diagnosis, effective abortive and preventive treatments of migraine are paramount to improve quality of life and as well as cost saving.

## Background

Migraine prevalence is highest during the productive years of individuals between the ages of 25 and 55 [1]. Migraine may cause significant impact on activity of daily living including physical and emotional functions [2]. Global Burden of Disease Survey (GBD) 2010 [3] estimated that the global prevalence of migraine is approximately 15% and migraine was ranked as third most common diseases in the world behind dental caries and Tension type headache (TTH). In 2013, migraine was recognized as the sixth-highest cause of disability in the world [4]. Migraine is a lifelong illness causing remarkable disability during the headache attacks, along with substantial financial burden to the patient as well as to the society. It also affects family and social relationships and impoverishes quality of life [5, 6]. In approximately 2% of the population, a chronic form of migraine became more evident resulting in a significant impact on daily activities [7]. Migraine prevalence in Saudi Arabia, Qatar and Oman, is within the estimated worldwide prevalence range of 0.7–21.9% [8]. The lifetime migraine prevalence in the three Gulf countries was lower than in European studies, which report a range of 12–28% [9]. Migraine disability imprints on Kuwaiti population were underreported since the GBD2010 [2] and GBD2013 [3] estimation for migraine were derived from limited data extracted from hospital-based studies. Hence, our study aimed to assess the prevalence of migraine and its burden in Kuwait through a comprehensive approach utilizing a door-to-door survey. In a prior publication, we reported on prevalence of primary headache disorders. In the present study, we further report on the prevalence of episodic migraine stratified by age and gender, characters of migraine in addition to its burden, which was not shown in our previous published paper [10].

## Methods

The full methodology was described elsewhere [10]. The fieldwork was carried out between January 2016 and April 2016. A population-based multistage random cluster sampling was conducted. Kuwaiti adults aged 18–65 years who were living in Kuwait for the last 6-month were identified. Adults who have deafness or major mental illness were excluded because of the communication difficulties with the interviewers. One adult member of each household was randomly selected after obtaining an informed consent. The interviewers consisted of 4 nurses and 10 medical students who were native Arabic speakers. The survey used several questionnaires such as Lifting The Burden (LTB) [11], Headache-Attributed Restriction, Disability, Social Handicap and Impaired Participation (HARDSHIP) questionnaire [12, 13] which were all translated into Arabic. Subjects were asked if all their headaches were of one or more types and, if more than one headache were identified, subsequent questions were focused on the one that was the most bothersome. The authors J.Y.A.-H. and S.F.A. established the diagnosis using an algorithm that was developed by LTB questionnaire [14] and based on ICD-II criteria [15]. Written informed consent was obtained from all the participants and they were free to decline participation at any time during the interviews.

### Data analysis

Data of completed questionnaires were entered into SPSS version 20.0. Collected data was analyzed to assess 1-year prevalence for migraine headache as percentages with 95% confidence intervals (CIs). Prevalence of migraine was stratified by gender and age. Estimates both genders according to age categories (18–30, 31–40, 41–50 and 51–65) were calculated. Categorical variables were described in terms of frequency and proportions (%, with 95% CIs where appropriate), continuous variables in terms of means and standard deviations (SDs). We used chi-square to compare distributions between categorical variables and t-test to compare between numerical variables. We used Pearson’ s coefficient to test for correlation between continuous variables. We considered a *P* value of <0.05 as statistically significant.

## Results

The interviewers collected 16,332 questionnaires, of which 15,523 were complete and subsequently used for analysis. The participation rate was 53.26%. The study sample presented 1.23% of the general population. A flowchart of the participants was published in our earlier paper [10]. Males represent 51.1% (*n* = 7925) of the analyzed sample. The mean age of the overall sample was 37.51 ± 11.82 years.

Among the total respondents, 23.1% (*n* = 3588) were diagnosed with episodic migraine while 5.4% (*n* = 845) were diagnosed as chronic headache. The 1-year prevalence of episodic migraine was significantly higher in female (31.71%) versus. Males (14.88%); *p* < 0.01. The majority of the migraine population was female (67%). The mean age of migraine subjects was significantly lower than subjects without migraine (34.56 ± 10.17 vs. 41.67 ± 12,94; *p* < 0.01). On the other hand, females with migraine were significantly younger than males with migraine (34.17 ± 10.70 vs. 35.43 ± 8.92; *p* < 0.01).

Table 1 shows the 1-year prevalence of migraine headache stratified by age and gender. Migraine prevalence is based on the reported most bothersome headache. Age specific prevalence of episodic migraine was higher in age group 18–30 (27%) compared to other age groups. Beyond 50 years of age, the prevalence fell in both genders (males: 0.5%; females 14%).

**Table 1:** One-year prevalence of Episodic Migraine Headache stratified by age and gender

Episodic Migraine	Total			Male			Female			*P*
Age (years)	Participant populations	Migraine cases	Prevalence % (CIs 95%)	Participant populations	Migraine cases	Prevalence % (CIs 95%)	Participant populations	Migraine cases	Prevalence % (CIs 95%)	
18–30	5056	1382	27.33 (17.48–28.89)	2762	415	15.05 (6.06–16.44)	2294	967	42.1 (30.8–58.4)	0.001^*^
31–40	4997	1292	25.86 (17.36–72.19)	2433	431	18.1 (6.4–19.70)	2564	861	32.93 (18.3–64.3)	0.02^*^
41–50	3198	752	23.54 (22.10–25.05)	1511	327	21.66 (19.60–23.82)	1687	425	25.18 (23.12–27.32)	0.43
51–65	2272	162	7.13 (6.11–8.27)	1219	6	0.5 (0.13–1.3)	1053	156	14.81 (12.11–17.84)	0.001^*^
Total	15,523	3588	23.11 (19.43–32.87)	7925	1179	14.88 (9.54–57.9)	7598	2409	31.70 (32.87–45.24)	0.01^*^

*CI* confidence interval

*Significant *P* Value

The characteristics of episodic migraine headache were summarized in Table 2. Mean days of headache in the last month in our study were 6.67. The majority (60%) had migraine headaches of moderate intensity. Most of migraine cohort (64.4%) sought advice of health services. Most of the migraine headache patients (62.4%) were seen by general practitioners (GPs) while neurologist, and other specialties saw 17.2% and 3.7% of patients respectively (*P* < 0.001). Only 4.6% of migraine cohort received a migraine diagnosis from a medical professional while, 8.9% was diagnosed as tension type headache and 2.1% were diagnosed as sinus headache. The majority (94.6%) of migraine subjects used symptomatic drugs for their headache attacks during the past month, whereas 39.9% were taking preventive medication at the time of the survey.

**Table 2:** Character of episodic migraine headache among Kuwaiti populations (*N* = 3588)

Variables	M ± SD/No (%)
Mean Age	34.56 ± 10.17
Marital state	
• Single	641 (17.9)
• Married	2738 (67.3)
• Divorced	100 (2.8)
• Widow	109 (3)
Education	
• University	2269 (63.2)
• High School	1164 (32.4)
• Primary School	17 (0.4)
Employment	
• Full time	2513(70)
• Part time	538 (15)
• Student	170 (4.7)
• Housewife	239 (6.7)
• Not working	128 (3.6)
Frequency of attacks/month	6.67 ± 2.33
Duration of attacks in hours	6.08 ± 334
Severity of headache	
• Not bad	35 (1.4)
• Quite bad	2170 (60.4)
• Very bad	1369 (38.2)
Treatments taken	
• Symptomatic treatment	3395 (94.6)
• Prophylactic treatment	1433 (39.9)
Use Health service	2309 (64.4)
Consultations	
• General practitioner	2253 (62.8)
• Neurologist	616 (17.2)
• Persons prescribing Traditional Medicine	586 (16.3)
• Other specialties	81 (2.3)

*M* Mean, *SD* standard deviation

There were significant positive correlations between both frequency of migraine attacks and lost days as shown in Table 3. The more frequent the attacks were, the more lost days observed in the preceding 3 months of the survey. However, the severity of attacks had only a negative impact on the social and family activities.

**Table 3:** Impact of migraine attacks assessed by HALT questionnaire in preceding 3 months

Variables	Lost workdays	Lost housework days	Lost social and family days
Frequency of attacks/month	*R* = 0.082 *P* = 0.001^*^	*R* = 0.125 *P* = 0.001^*^	*R* = 0.141 *P* = 0.001^*^
Severity of migraine attacks	*R* = 0.029 *P* = 0.098	*R* = 0.022 *P* = 0.181	**R** = 0.047 *P* = 0.005^*^

HALT índex headache-attributed lost work, housework and social days

*Significant *P* Value

The cumulative number of lost days according to the HALT index was outlined in Table 4. Subjects with chronic headaches had a significantly higher burden. Subjects with episodic migraine had lost a mean of 1.97 days from their paid work or school attendance, versus 6.62 lost days in patients with chronic headache (*p* < 0.001). Similarly, subjects with episodic migraine lost a mean of 1.40 days from their household work versus 5.35 lost days in subjects with chronic headache (*p* < 0.001) in the preceding 3 months of the survey. Regarding social and family activities, subjects with episodic migraine and chronic headache missed mean of 2.81 and 3.85 days in the preceding 3 months of the survey (*P* < 0.001).

**Table 4:** Impact of episodic migraine versus chronic headache assessed by HALT questionnaire in preceding 3 months

Variables	Episodic Migraine M ± SD	Chronic Headache M ± SD	*P* value
Work days	1.87 ± 1.71	6.62 ± 2.41	0.001^*^
Housework days	1.44 ± 1.83	5.35 ± 2.36	0.001^*^
Social and family days	2.81 ± 1.75	3.85 ± 2.95	0.001^*^

*HALT índex* headache-attributed lost work, housework and social days, *M* Mean, *SD* standard deviation

*Significant *P* Value

## Discussion

The study captured data from all six governmental sectors of Kuwait. The 1-year prevalence of migraine in our study was 23%, which was higher than previous studies conducted in Arab Gulf countries (Qatar 7.9% [16] and in Oman 10.1% [17]). Our result was similar to the prevalence for other international figures of 22% in Georgia [18], 25.2% in Pakistan, [19], 20.8% in Russia [20] 25.6 in India [21], 22.9% in Zambia [22], and 29% in Turkey [23]. On the other hand, our result was higher than that of the global estimate (14.7%) [2] other countries such as Colombia (13.7%) [24], Germany (18%) [25] and China study (9.3%) [26]. In Italy, migraine 1-year prevalence was 42.9%; which is much higher than our result (5) and also the 1-year prevalence of migraine in Eurolight project 35.3% [27] The discrepancies in the prevalence figures could be attributed to methodological differences, and to genetic, environmental or cultural factors. Our estimation of migraine prevalence in the Kuwaiti population is matching to our previous estimation of migraine among medical students (27.9%) [28]. Our study reported a female preponderance of 67% that could be hormonally determined which is consistent with previous studies [29].

More than 38% of migraine subjects were younger than 30 years of age and approximately 68% were in the age group between 18 to 50 years of age similar to previous published studies [19, 20, 23]. The highest age specific prevalence of episodic migraine in our cohort is 27.3% in the age group 18–30 years and the lowest prevalence is in the age group 51–65 years. Prevalence of migraine in females is high in the 18–30 age group and middle age group (31–40 years) but sharply declined in the postmenopausal age group (51–65 years), which is similar to previous reports [20, 24]. These trends can be attributed to the well-documented influence on migraine of estrogen levels [29]. However migraine prevalence declined also in our male cohorts older than 50 years.

Most of migraine cohort sought medical advice for their headaches. Although the majority was seen by their GPs, only 4.6% received accurate diagnosis of migraine. A study conducted in the UK [30] indicated that 70% of migraine headache patients were not given a diagnostic label, by GPs. It was suggested that GPs’ difficulty in diagnosing migraine headache presentations results in increase in the impact of migraine headaches on activity of daily living. [31]. Interestingly, in our study, migraine cohort showed that seeking traditional Arab treatment, like; Hijama is nearly equal to seeking neurologist. A significant number of our population continues to believe in this type of treatment, which is performed by non-medical personnel. The community awareness with migraine needs to be improved and the education of the primary care providers is an important step in this aspect to limit the numbers of under-diagnosed or misdiagnosed patients. This high percentage of patients who sought medical advice reflects the impact of migraine on health resources. Previous studies reported that headache was one of the highest indications seen by general practitioners and neurologists [32, 33]. A survey of neurologists indicated that up to a third of all their referral were related to headaches [34]. We reported that 94.6% of our cohort had used acute medication, which was nearly similar to an Austrian study, which reported that 94.4% had used acute abortive medications. Similarly, the proportion of patients who used prophylactic medication in our study (39.9%) was comparable to the Austrian study (34%) [35].

Despite the significant burden of migraine in different countries, the impact of migraine on daily activities was poorly described in Kuwait. We reported that subjects with episodic migraine lost an average 1.8 days from their paid work in the preceding 3 months to the survey, which was nearly similar to an Italian study of 2.3 lost days [5] and Eurolight project that reported mean lost workdays about 1 day/ month [27]. The frequency of migraine attacks in our cohort was significantly correlated with missing working, school and social days, and household activities during their migraine attack. Our results were in line with previous findings of a direct correlation between attack frequency and migraine-related disability for people with migraine [36].

Social activity and work capacity were reduced in almost all people with migraine [37]. Our results reported chronic headache in 5.4% of the total cohort. We demonstrated that the rate of disability days per month (using HALTquestionnaire) was significantly higher in chronic headache participants compared to episodic migraine participants. This is consistent with previous studies [35, 36, 38–40]. Thus, chronic headache participants were less able to perform work and leisure activities than those with episodic migraine participants. Chronic headache might affect family and social life along with employment [37, 38].

Since migraine is most troublesome in the productive years, its public health importance lies in its causal association with these personal and societal burdens of pain, disability, damaged quality of life and financial cost [40]. Many governmental and nongovernmental stakeholders do not recognize that the direct costs of migraine management is smaller when compared with the large indirect-cost savings that might be made from reducing lost working days, school days, social activities [5, 26, 41, 42].

One major limitation that needs to be outlined is the sampling methodology. Although we propose a probability sample method (cluster sampling), it is not adjusted for gender and age according to the participation of the Kuwaiti population; hence it may not entirely reflect the distribution of the whole population. To minimize this potential source of bias, we used a large sample.

Another limitation was the recall bias, which is usual in most of the studies using questionnaire. On the other hand, this study is the first and largest population-based survey in Kuwait to estimate the prevalence of migraine headache, and the first to measure migraine-attributed burden.

## Conclusions

Migraine is prevalent in Kuwait and has a significant socioeconomic burden especially in its chronic form. Improving the public awareness of migraine through health education of primary care providers and public media may reduce the misdiagnosis and subsequently result in better quality of life and reduce the economic burden.

## Abbreviations

CIs: Confidence intervals
GBD: Global Burden of Disease Survey
GPs: General practitioners
HALT: Headache-Attributed Lost Time Index questionnaire
HARDSHIP: Headache-Attributed Restriction, Disability, Social Handicap and Impaired Participation questionnaire
LTB: Lifting The Burden
SDs: Standard deviations
TTH: Tension type headache
